# A finite element model for protein transport in vivo

**DOI:** 10.1186/1475-925X-6-24

**Published:** 2007-06-28

**Authors:** Kouroush Sadegh Zadeh, Howard C Elman, Hubert J Montas, Adel Shirmohammadi

**Affiliations:** 1 Department of Computer Science, University of Maryland, College Park, MD, 20742, USA; 2 Fischell Department of Bioengineering, University of Maryland, College Park, MD, 20742, USA

## Abstract

**Background:**

Biological mass transport processes determine the behavior and function of cells, regulate interactions between synthetic agents and recipient targets, and are key elements in the design and use of biosensors. Accurately predicting the outcomes of such processes is crucial to both enhancing our understanding of how these systems function, enabling the design of effective strategies to control their function, and verifying that engineered solutions perform according to plan.

**Methods:**

A Galerkin-based finite element model was developed and implemented to solve a system of two coupled partial differential equations governing biomolecule transport and reaction in live cells. The simulator was coupled, in the framework of an inverse modeling strategy, with an optimization algorithm and an experimental time series, obtained by the Fluorescence Recovery after Photobleaching (FRAP) technique, to estimate biomolecule mass transport and reaction rate parameters. In the inverse algorithm, an adaptive method was implemented to calculate sensitivity matrix. A multi-criteria termination rule was developed to stop the inverse code at the solution. The applicability of the model was illustrated by simulating the mobility and binding of GFP-tagged glucocorticoid receptor in the nucleoplasm of mouse adenocarcinoma.

**Results:**

The numerical simulator shows excellent agreement with the analytic solutions and experimental FRAP data. Detailed residual analysis indicates that residuals have zero mean and constant variance and are normally distributed and uncorrelated. Therefore, the necessary and sufficient criteria for least square parameter optimization, which was used in this study, were met.

**Conclusion:**

The developed strategy is an efficient approach to extract as much physiochemical information from the FRAP protocol as possible. Well-posedness analysis of the inverse problem, however, indicates that the FRAP protocol provides insufficient information for unique simultaneous estimation of diffusion coefficient and binding rate parameters. Care should be exercised in drawing inferences, from FRAP data, regarding concentrations of free and bound proteins, average binding and diffusion times, and protein mobility unless they are confirmed by long-range Markov Chain-Monte Carlo (MCMC) methods and experimental observations.

## Background

Transport of mass, energy, and momentum has a crucial role in many branches of science and engineering. In biological systems, transport phenomena are central to the biological processes that take place in different parts of organisms. They determine the behavior and function of cells, tissues, and organs, and regulate interactions between synthetic agents (e.g. drugs) and recipient targets. These phenomena are crucial elements in the design and use of biosensors, high density cell culture, filtration units for kidney dialysis, heart-lung bypass machines, and membrane oxygenators in human medical care and ion selective electrodes, pH-meters, electrical conductivity meters, and time domain reflectometery used in biosystem analysis. Transport processes are critical in the removal of toxins from the blood, remediation of impaired water bodies (sources of waterborne diseases), and bioremediation of contaminated landscape [1,2].

Fluorescence Recovery after Photobleaching (FRAP) is one of the most widely used experimental protocols to study biological transport processes such as diffusion and reaction [3-30]. FRAP is a straightforward technique used to monitor the movement of fluorescence molecules. These molecules can absorb light of one wavelength (blue for instance) and emit light of another (e.g. green). However, if exposed to repeated cycles of excitation-emission, they lose their ability to emit fluorescence. This phenomenon is called *"photobleaching" *or *"photochemical bleaching" *[24]. In this technique a small region of living cell containing Green Fluorescent Protein (GFP)-tagged protein is exposed to a brief but intense laser beam, produced by a laser scanning confocal microscope, to irreversibly inactivate fluorescence emission in that region. Before exposure to the light, the living cell is in equilibrium with a uniform population of fluorescence [25]. Photobleaching creates two different populations of fluorescence molecules, which are spatially separated at the beginning of the experiment. Unbleached molecules from the undisturbed area move toward the bleached region and the rate of fluorescence recovery is measured as a function of time. The result is a noisy graph known as a recovery curve. However, because of the noise, the original graph by itself is not suitable for quantitative study of the dynamics of living cells. The FRAP community generally uses a data processed normalized average fluorescence recovery curve that has less noise. By analyzing the recovery curve, one can quantify how many fluorescent photons return to the bleached area in comparison to the amount of light that was there before photobleaching. This is known as *percent recovery*. The other question that can be addressed is that of how fast the fluorophores move toward the bleached area. This is a measure of the *free molecular diffusion coefficient *of the bio-macromolecule under study.

One of the first attempts to estimate bio-macromolecule mass transport and binding rate parameters using in vivo information was carried out by Kaufmann and Jain [13]. Sprague et al. [25] developed a diffusion-reaction model to simulate FRAP experiment but the solution is in Laplace space and requires numerical inversion to return to real time. The model presented recently by Lele et al. [26] properly respects cell boundaries but is in the form of a Fourier-Bessel series and can suffer from Gibbs phenomena. Carrero et al. (2003, 2004) presented an excellent review on the effects of boundary conditions, influence of the membrane, and the location of the photobleaching on the estimation of diffusion coefficients for diffusing biomolecules in a bounded domain [24,28]. They showed that overestimations or underestimations can result from ignoring this influence [24]. Beaudouin et al. [29] used a diffusion-reaction models to study mobility of five chromatin-interacting proteins inside living cells. They found that transient interactions are common for chromatin proteins. Individual proteins locally sample chromatin for binding sites rather than diffusing globally followed by binding at random nuclear positions. They concluded that complementary procedures are needed to measure transient biochemical interactions in living cells.

Although experimental methods are representative of the biological system, they are expensive, tedious and time-consuming. An alternative approach is to use mathematical modeling. In this regard, several sophisticated mathematical models have been developed to predict and simulate the fate and transport of drugs and bio-macro-molecules in biological systems. However, the use of these models is not an easy task since they contain numerous parameters that need to be determined before the model(s) can be used for the considered situation. The success of model predictions depends largely on the proper representation of relevant processes, uncertainty in model parameters [31,32], and parameter identification which is a critical step in the modeling process. Difficulties in model calibration and parameter identification are quite common in modeling mass transport problems in biological systems [1,30].

The main objective of this paper is to develop a mass-lumped Galerkin-based finite element model (FEM) to solve a system of two partial differential equations governing protein transport and binding in living cells and couple it with the Osborne-Moré [33,34] extended version of the Levenberg-Marquardt [35,36] algorithm and an experimental data set, obtained by the Fluorescence Recovery after Photobleaching (FRAP) technique, to quantify bio-macromolecule diffusion coefficient and binding rate parameters. The applicability of the developed FEM-based inverse modeling strategy is illustrated by simulating the mobility and binding of GFP-tagged glucocorticoid receptor in the nucleoplasm of mouse adenocarcinoma.

## Methods

### Direct problem

A one site-mobile-immobile model was selected as the *direct (forward) problem *to describe bio-macromolecular diffusion inside living cell in cylindrical coordinate system [1,30]:

(1) $$\begin{array}{l} \frac{\partial F}{\partial t}(\rho ,t)=D_{F}\frac{\partial^{2}F}{\partial \rho^{2}}(\rho ,t)+D_{F}\frac{1}{\rho }\frac{\partial F}{\partial \rho }(\rho ,t)-K_{a}^{*}F(\rho ,t)+K_{d}C(\rho ,t)\\ \frac{\partial C}{\partial t}(\rho ,t)=K_{a}^{*}F(\rho ,t)-K_{d}C(\rho ,t) \end{array}$$

Since a circular bleach spot was used to bombard the cell and to track the fluorescently tagged biomolecules inside bleach spot during the time course of a FRAP experiment, system (1) was written in cylindrical coordinate system.

Furthermore, the binding reaction was modeled by primary rate kinetic or single binding site model [1,13,25,26,30]:

(2) $$\text{F}+\text{S}\overset{{\text{K}}_{\text{a}}}{\underset{{\text{K}}_{\text{d}}}{⇄}}\text{C}$$

subject to the following initial and Neumann boundary conditions:

$$\begin{array}{l} \begin{array}{cc} F(\rho ,0)=\{\begin{array}{l} 0,\\ F_{eq}, \end{array} & \begin{array}{c} 0<\rho \leq w\\ w<\rho \leq R \end{array} \end{array}\\ \begin{array}{cc} C(\rho ,0)=\{\begin{array}{l} 0,\\ C_{eq}, \end{array} & \begin{array}{c} 0<\rho \leq w\\ w<\rho \leq R \end{array} \end{array}\\ {\frac{\partial F}{\partial \rho }|}_{\rho =0,R}=0\\ {\frac{\partial C}{\partial \rho }|}_{\rho =0,R}=0 \end{array}$$

where *F *is the concentration of free biomolecule, *S *is the concentration of vacant binding sites, *C *is the concentration of the bound complex(*C *= *FS*), *D*_*F *_is the molecular diffusion coefficient (*L*^2^*T*^-1^) of free biomolecule, *K*_*a *_is the free biomolecule-vacant binding site association rate coefficient (*T*^-1^), *K*_*d *_is dissociation rate coefficient (*T*^-1^), $K_{a}^{*}$ = *K*_*a*_*S *is the pseudo-association rate coefficient, *ρ *is radial coordinate (*L*) in the cylindrical coordinate system, *w *is the radius of the bleached area, *R *is the length of the spatial domain, and *F*_*eq *_and *C*_*eq *_are equilibrium concentration of *F *and *C *[25,30]:

$$\begin{array}{cc} F_{eq}=\frac{K_{d}}{K_{a}^{*}+K_{d}}, & C_{eq}=\frac{K_{a}^{*}}{K_{a}^{*}+K_{d}} \end{array}$$

The initial condition implies that photochemical bleaching inactivates the fluorescence tag on the biomolecules in the bleached area but does not change the concentrations of free and bound biomolecules or vacant binding sites. The boundary conditions imply that the diffusive biomolecule flux is zero at the center of the bleach spot and at the cell or nucleus membrane during time course of a FRAP experiment [30].

The forward problem was solved by the finite element method. The weak formulation of the dependent variables *F *and *C *were approximated using piecewise linear approximating functions [1,31,37-39]:

(3) $$\begin{array}{c} F^{'}(\rho ,t)=\sum_{j=1}^{N}F_{j}(t)\varphi_{j}(\rho )\\ C^{'}(\rho ,t)=\sum_{j=1}^{N}C_{j}(t)\varphi_{j}(\rho ) \end{array}$$

in which *N *is total number of nodes in the spatial domain, *ϕ*_*j*_(*ρ*) are the selected linear basis functions, and *F*_*j*_(*t*) and *C*_*j*_(*t*) are the associated time-dependent unknown coefficients that represent the solution of Equation (1) at nodes within the domain.

Substitution of expressions (3) into Equation (1) will not satisfy the partial differential equation and hence will produce a residual. The goal of the finite element approximation is to minimize this error. This can be accomplished by introducing the weight function, *ϕ*_*i*_(*ρ*), and setting the integral of the weighted residuals to zero. In the Galerkin method, which was used in this study, the weighting functions are chosen to be identical to the basis function [1,31]:

(4) $$\begin{array}{l} \int_{\Omega }\left[\frac{\partial F^{'}}{\partial t}(\rho ,t)-D_{F}\frac{\partial^{2}F'}{\partial \rho^{2}}(\rho ,t)-D_{F}\frac{1}{\rho }\frac{\partial F'}{\partial \rho }(\rho ,t)+K_{a}^{*}F'(\rho ,t)-K_{d}C'(\rho ,t)\right]\varphi_{i}(\rho )d\Omega =0\\ \int_{\Omega }\left[\frac{\partial C'}{\partial t}(\rho ,t)-K_{a}^{*}F'(\rho ,t)+K_{d}C'(\rho ,t)\right]\varphi_{i}(\rho )d\Omega =0 \end{array}$$

where Ω is the study domain. Applying Green's first identity [37,39] to equation (4) yields:

(5) $$\begin{array}{l} \sum_{e}\int_{\Omega_{e}}\frac{\partial F^{'}}{\partial t}(\rho ,t)\varphi_{i}(\rho )d\Omega +D_{F}\sum_{e}\int_{\Omega_{e}}\frac{\partial F'}{\partial \rho }(\rho ,t)\frac{\partial \varphi_{i}(\rho )}{\partial \rho }d\Omega -D_{F}\sum_{e}\int_{\Omega_{e}}\rho \frac{\partial F'}{\partial r}(\rho ,t)\varphi_{i}(\rho )d\Omega \\ +K_{a}^{*}\sum_{e}\int_{\Omega_{e}}F'(\rho ,t)\varphi_{i}(\rho )d\Omega -K_{d}\sum_{e}\int_{\Omega_{e}}C'(\rho ,t)\varphi_{i}(\rho )d\Omega =-q_{fn}\\ \sum_{e}\int_{\Omega_{e}}\frac{\partial C^{'}}{\partial t}(\rho ,t)\varphi_{i}(\rho )d\Omega -K_{a}^{*}\sum_{e}\int_{\Omega_{e}}F'(\rho ,t)\varphi_{i}(\rho )d\Omega +K_{d}\sum_{e}\int_{\Omega_{e}}C'(\rho ,t)\varphi_{i}(\rho )d\Omega =-q_{cn} \end{array}$$

in which Ω_*e *_is the domain of element and *q*_*fn *_and *q*_*cn *_are fluxes of the free and bound bio-macromolecules across the boundary out of the element, respectively.

The time derivatives in equation (5) were approximated using an Euler time-marching algorithm (backward finite difference scheme). Inserting equation (3) into equation (5) and integrating over the elements produces a system of time-dependent ordinary differential equations which can be formulated in matrix form:

(6) $$\begin{array}{l} {}[A]\frac{{\left\{F\right\}}^{n+1}-{\left\{F\right\}}^{n}}{\Delta t}+[B]{\left\{F\right\}}^{n+1}+[E]{\left\{F\right\}}^{n+1}-[H]{\left\{C\right\}}^{n+1}={\left\{Q_{f}\right\}}^{n+1}\\ {}[A]\frac{{\left\{C\right\}}^{n+1}-{\left\{C\right\}}^{n}}{\Delta t}-[E]{\left\{F\right\}}^{n+1}+[H]{\left\{C\right\}}^{n+1}={\left\{Q_{c}\right\}}^{n+1} \end{array}$$

where:

$$\begin{array}{l} {}[A_{1}]=\sum_{e}\int_{\Omega_{e}}\varphi_{i}\varphi_{j}d\Omega \\ {}[B]=\sum_{e}D_{f}\int_{\Omega_{e}}\frac{\partial \varphi_{i}}{\partial \rho }\frac{\partial \varphi_{j}}{\partial \rho }d\Omega -\sum_{e}\frac{D_{f}}{\rho_{e}}\int_{\Omega_{e}}\frac{\partial \varphi_{i}}{\partial \rho }\varphi_{j}d\Omega \\ {}[E]=\sum_{e}K_{a}^{*}\int_{\Omega_{e}}\varphi_{i}\varphi_{j}d\Omega \\ {}[H]=\sum_{e}K_{d}\int_{\Omega_{e}}\varphi_{i}\varphi_{j}d\Omega \\ \left\{Q_{f}\}=\{0,0,.........,0,q_{fn}\right\}\\ \left\{Q_{c}\}=\{0,0,.........,0,q_{cn}\right\} \end{array}$$

in which *ρ*_*e *_is the radial position of the centroid of element *e*.

### Parameter optimization

The inverse problem was formulated as a nonlinear optimization problem in which model parameters *p *= [*D*_*f*_, $K_{a}^{*}$, *K*_*d*_] were optimized by minimizing a penalty function representing the discrepancy between the observed and predicted average fluorescence intensity recovery time series inside the bleach spot [1]. If the measurement errors asymptotically follow a multivariate normal distribution with zero mean and covariance matrix,*V*, the likelihood function can be formulated as [40]:

(7) $$L(p)={\left(2\pi \right)}^{-N/2}\det{\left[V\right]}^{-1/2}\exp[-\frac{1}{2}{\left(U^{*}-U(p)\right)}^{T}V^{-1}(U^{*}-U(p))]$$

where *L*(*β*) is the likelihood function, *N *is the number of observations, *p *is the vector of the parameters being optimized, U* is a vector and/or matrix of observations, and *U *is a corresponding vector and/or matrix of model predictions as a function of the parameters being optimized. This vector is obtained by solving the forward problem. The maximum likelihood estimator consists of those values of the unknown parameters that maximize the magnitude of the same likelihood function [40]. Since logarithm is a monotonically increasing function of its argument (the value of *p *that maximizes *L*(*p*) also maximizes ln *L*(*p*)), and because ln *L*(*p*) is simpler and much easier to use than *L*(*p*) itself, therefore ln *L*(*p*) is usually used in optimization:

(8) $$\ln L(p)=-\frac{N}{2}\ln(2\pi )-\frac{1}{2}\det[V]-\frac{1}{2}{\left(U^{*}-U(p)\right)}^{T}V^{-1}(U^{*}-U(p))]$$

The maximum of the likelihood function must satisfy the set of equations *∂ *ln *L*(*p*)/*∂p *= 0. When the error covariance matrix is known, maximization of equation (8) is equivalent to the minimization of the following weighted least square problem [1]:

(9) *ϕ*(*p*) = [(U* - *U*(*p*))^*T*^*V*^-1^(U* - *U*(*p*))]

where *ϕ*(*p*) is the objective or penalty function. If there is information about the values and distributions of parameters, it can be incorporated in the objective function as well [1]:

(10) $$\varphi (p)=[{\left(U^{*}-U(p)\right)}^{T}V^{-1}(U^{*}-U(p))]+[{\left(p^{*}-\hat{p}\right)}^{T}V_{p}^{-1}(p^{*}-\hat{p})]$$

in which p* is parameter vector containing a priori information, $\hat{p}$ is the corresponding predicted parameter vector, and *V*_*p *_is the covariance matrix for the parameter vector. This kind of optimization is known as *Bayesian estimation*. The second term in equation (10), which is sometimes called the *plausibility criterion *[1] insures that the optimized values of the parameters remain in some feasible region around p*. Matrices *V *and *V*_*p*_, which are sometimes called weighting matrices, provide information about the measurement accuracy as well as any possible correlation between measurement errors and between parameters.

A limitation of equation (10) is that the error covariance matrix is generally not known. A common approach to overcoming this problem is to make some a priori assumptions about the structure of the error covariance matrix. In the absence of any additional information regarding the accuracy of input data, the simplest and most recommended way is to assume that observation errors are uncorrelated which implies setting *V *equal to the identity matrix and *V*_*p *_to zero. In this case the optimization problem collapses to the well known ordinary least squares formulation [41,42]:

(11) $$\varphi (p)=\frac{1}{2}r{\left(p\right)}^{T}r(p)$$

where *r *is the residual (differences between the observed and predicted state variable) column vector.

Minimization of equation (11) was carried out iteratively by first starting with an initial guess of parameter vector, {*p*^(*k*)^} and updating it at each iteration until the termination criteria were met [1,31,32]:

(12) *p*^(*k*+1) ^= *p*^(*k*) ^+ *α*^(*k*)^Δ*p*^(*k*)^

where *α*^(*k*) ^is a scalar step length and Δ*p*^(*k*) ^is the direction of the search or step direction [41].

Using *QR *decomposition [43] the linear least square problem below, which is the Osborne-Moré extended version of the Levenberg-Marquardt algorithm, was solved to obtain the search direction in each iteration:

(13) $$\min{\left\|\left(\begin{array}{l} r(p^{k})\\ 0 \end{array}\right)+\left(\begin{array}{l} J{\left(p\right)}^{k}\\ {\left(\lambda^{k}D^{k}\right)}^{\frac{1}{2}} \end{array}\right)\Delta p^{k}\right\|}^{2}$$

where *λ *is a positive scalar known as Marquardt's parameter or Lagrange multiplier [36], *J *is the Jacobian or sensitivity matrix, and *D *is a positive definite scaling matrix that ensures the descent property of the algorithm even if the initial guess is not "smart". For non-zero values of *λ*, the Hessian approximation is always a positive definite matrix, which ensures the descent property of the algorithm [41].

A combination of "one-sided" and "two-sided" finite difference methods [30-32] was used to calculate the partial derivatives of the state variable with respect to model parameters and to construct the Jacobian matrix. The "One-sided" finite difference method estimates the partial derivatives of the state variable with respect to model parameters by solving the forward problem (Eq. 1) ***p+1 ***times (***p ***is number of parameters to be estimated). On the other hand, the "two-sided" finite difference method estimates the partial derivatives of the state variable with respect to model parameters by solving the forward problem (Eq. 1) 2***p+1 ***times. At the early stages of the optimization, where the search is far from the solution, the "one-sided" finite difference scheme, which is computationally cheap but not as accurate as the "two-sided" approach, was used. As the optimization proceeds in the descent direction, the algorithm switches to a more accurate but computationally expensive approach in which the partial derivatives of the state variable with respect to the model parameters are calculated using a two-sided finite difference scheme. The switch was made when *ϕ*(*p*) ≤ 1 × 10^-2^. A detailed description of the procedure to update the Jacobian matrix is presented in [1,30].

In order to update *λ *at each iteration, the optimization starts with an initial parameter vector and a large *λ*(*λ *= 1). As long as the objective function decreases in each iteration, the value of *λ *is reduced. Otherwise, it is increased. The approach avoids calculation of *λ *and step length in each iteration and is therefore computationally cheap. A detailed description of the code for updating *λ *is given in [31].

Finally, the following stopping rule was used to end the search [1,31,32]:

$$if\;({\nabla \varphi (p)|}_{p=\hat{p}}\leq \delta_{1}\&\frac{\Delta \varphi (p)}{\varphi (p)}\leq \delta_{2}\&\varphi (p)\leq \delta_{3})$$

    *Stop*

else

    *Continue Optimization Loop*

end

where ${\nabla \varphi (p)|}_{p=\hat{p}}$ is the gradient of the penalty function at solution, $\frac{\Delta \varphi (p)}{\varphi (p)}$ is relative changes in the magnitude of the parameters in two consecutive iterations, and *δ*_1_, *δ*_2_, and *δ*_3 _are user defined small values.

The accuracy of the optimization was assessed by goodness-of-fit analysis. The Root Mean Squared Error (*RMSE*) and Coefficient of Determination (*R*^2^) were calculated for every set of optimized parameters [44,45]:

(14) *RMSE *= (*r*^*T*^*r*/(*N *- *p*))^1/2^

(15) $$R^{2}=\frac{{\left[\sum {\hat{I}}_{i}I_{i}-\sum {\hat{I}}_{i}\sum {\hat{I}}_{i}\right]}^{2}}{\left[\sum {\hat{I}}^{2}-{\left(\sum {\hat{I}}_{i}\right)}^{2}][\sum I^{2}-{\left(\sum I_{i}\right)}^{2}\right]}$$

where *I*_*i *_and ${\hat{I}}_{i}$ are the observed and predicted total normalized average fluorescence intensity, *F *+ *C*, inside bleached area during time course of FRAP experiment, respectively and *N *is the number of observations on FRAP time series.

## Experimental Studies

A FRAP experiment was conducted on the mouse adenocarcinoma cell line 3617 at the Laboratory of Receptor Biology and Gene Expression, National Cancer Institute-National Institutes of Health, Bethesda, MD (McNally, personal communication). This data set consists of 43 fluorescent recovery values gathered in the course of a 20-second FRAP experiment and post processed to reduce noise. The developed inverse modeling strategy was then used to quantify mass transport and binding rate parameters of GFP-tagged glucocorticoid receptor.

## Results and Discussion

### Model validation

**Figure 1:**
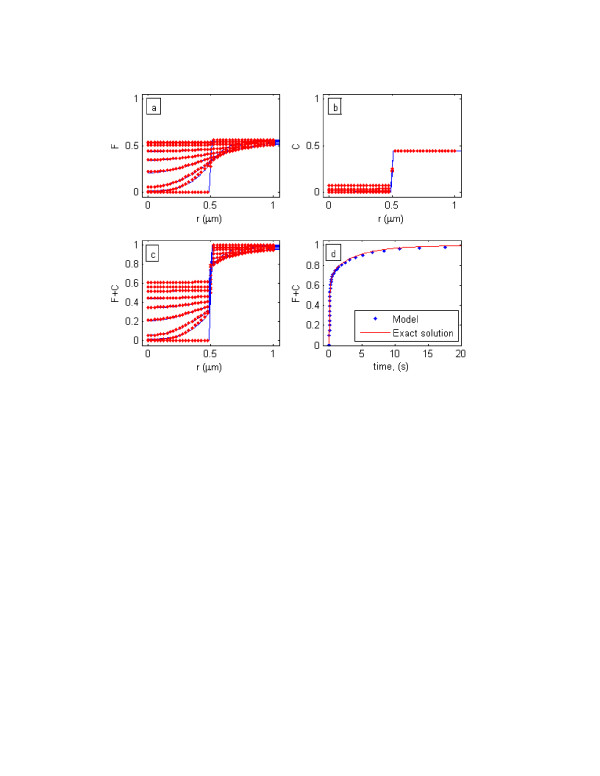
Spatial and temporal distributions of GFP-GR inside bleach spot after photo-chemical bleaching during time course of a FRAP experiment. The Figure shows comparison of the analytic solution (solid lines) and numerical model (dots) at times of 0, 0.01, 0.02, 0.05, 0.1, 0.2, 0.5, 1, and 2 seconds for free GFP-GR (a), bound complex (b), and total GFP-GR (c). Validation of the numerical model (dots) with the analytic solution (solid lines) of [25] is depicted in (d). The graph presents average normalized fluorescent intensity, obtained by equation (16), inside the bleach spot.

Before being incorporated into the framework of the developed inverse modeling strategy, the numerical model was first validated against the exact solution of Sadegh Zadeh and Montas (unpublished) and the semi-analytic solution of [25]. The results are depicted in Figures 1a, 1b, and 1c (for the exact solution of Sadegh Zadeh and Montas) and in Figures 1d (for the solution of [25]). To validate our model with the solution of [25], the average of the normalized fluorescence intensity within the bleach spot during the time course of the FRAP experiment, *I*(*t*), was calculated by [1,30]:

(16) $$I(t)=\frac{2}{w^{2}}\int_{0}^{w}\rho [F(\rho ,t)+C(\rho ,t)]d\rho$$

While Figure 1a shows spatial and temporal distributions of free GFP-GR, *F*(*ρ*, *D*_*f*_, $K_{a}^{*}$, *K*_*d*_, *t*), inside and outside bleach spot after photochemical bleaching during time course of a FRAP experiment, Figure 1b presents distributions of bound protein, *C*(*ρ*, *D*_*f*_, $K_{a}^{*}$, *K*_*d*_, *t*), at times of 0, 0.01, 0.02, 0.05, 0.1, 0.2, 0.5, 1, and 2 seconds. Both Figures indicates that there is excellent agreement between the analytic solution and numerical simulator. Spatial and temporal distributions of total fluorescently tagged GFP-GR, [*F *+ *C*](*ρ*, *D*_*f*_, $K_{a}^{*}$, *K*_*d*_, *t*), inside and outside the bleached area are given in Figure 1c, which presents excellent agreement between the analytic and numerical solutions. The same time range was used to perform the comparison. As Figure 1d indicates, there is excellent agreement between the two solutions in simulating the average normalized fluorescence intensity within the bleach spot during time course of the FRAP experiment.

### Model calibration

**Figure 2:**
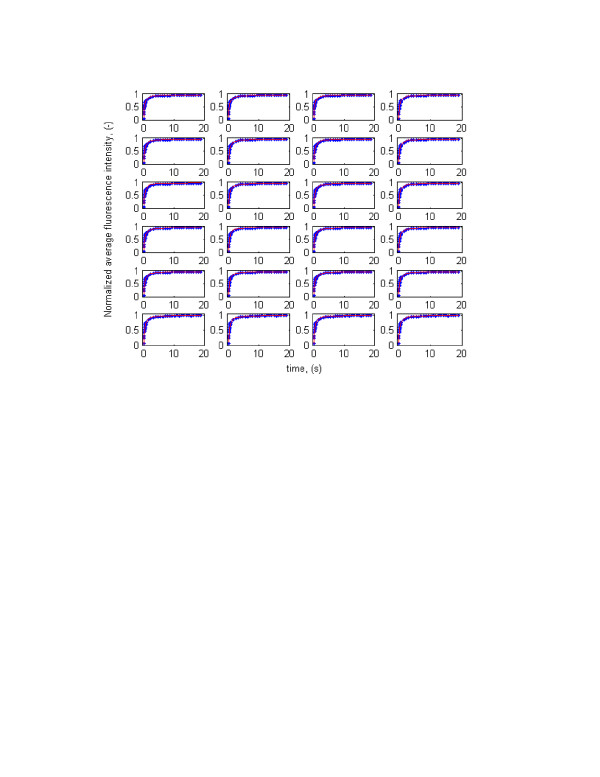
Predicted and experimental normalized average fluorescent intensity recovery curves for GFP-GR (dots: Observed, solid lines: Simulated) obtained by equation (16). The experimental data are from J. McNally (personal communication).

The developed methodology was then used to estimate diffusion coefficient and binding rate parameters of GFP-tagged glucocorticoid receptor (GFP-GR). The results are given in Table 1 and Figure 2 (the experimental FRAP time series data are from McNally, personal communication). The Root Mean Squared Error (*RMSE*) and Coefficient of Determination (*R*^2^) were calculated, using equations (14) and (15), for every set of optimized parameters and presented in the last two columns of Table 1. The values for diffusion coefficient, binding rate parameters, and corresponding indices estimated by [25] are given as the first run in Table 1 and Figure 2 for sake of comparison. Table 1 and Figure 2 indicate that many combinations of the three parameters can essentially produce the same error level (*RMSE*) and yields equally excellent fits. The values obtained by [25] represent only one of the possible solutions. In other words, the inverse problem is not well-posed and has no unique solution. Therefore, one may conclude that the Fluorescence Recovery after Photobleaching technique, though useful in studying the dynamics of biological systems, provide insufficient information to uniquely estimate free diffusion coefficient and binding rate parameters of biomolecule simultaneously.

**Table 1:** The results of parameter estimation for GFP-GR using an experimental time series obtained by the Fluorescence Recovery after Photobleaching (FRAP) technique.

run	*D*_*f *_(*μ m*^2^*s*^-1^)	$K_{a}^{*}$ (*s*^-1^)	*K*^*d *^(*s*^-1^)	*F*_*eq*_	*C*_*eq*_	*t*_*b *_(*ms*)	*t*_*d *_(*ms*)	*RMSE*	*R*^2^
1*	9.20	500	86.4	0.1473	0.8527	11.60	2	0.0255	0.9886
2	1.9049	1.0549	3.7657	0.7812	0.2188	265	0.948	0.0259	0.9897
3	1.2319	0.1113	12.0951	0.9909	0.0091	83	8985	0.0245	0.9903
4	3.4980	10.2501	8.3917	0.4502	0.5498	119	97.6	0.0275	0.9882
5	22.8472	369.7719	24.1606	0.0613	0.9387	41	2.7	0.0251	0.9898
6	81.9332	4785	75.4057	0.0155	0.9845	13	0.2	0.0233	0.9912
7	1.2160	0.7076	5057	0.9999	0.0001	0.2	1413	0.0245	0.9903
8	1.8034	0.5172	2.4778	0.8273	0.1727	403	1933	0.0259	0.9896
9	4.6451	35.3378	15.8084	0.3091	0.6909	63	28.3	0.0257	0.9895
10	4.4471	1.4531	2.0004	0.5792	0.4208	500	688	0.0321	0.9855
11	1.2014	0.00003	20.3473	1.0000	0.0000	49	3.3 × 10^7^	0.0246	0.9901
12	20.3662	928	62	0.0626	0.9374	16	1078	0.0233	0.9912
13	7.4662	101	23.3243	0.1876	0.8124	43	9901	0.0247	0.9904
14	1.2210	7.5124	1423	0.9947	0.0053	0.7	1333113	0.0245	0.9903
15	19.5330	160	13.9388	0.0801	0.9199	72	6250	0.0290	0.9865
16	1.2350	32.2818	1590	0.9801	0.0199	0.6	30977	0.0245	0.9902
17	11.0675	300.4231	40.4005	0.1185	0.8815	25	3329	0.0235	0.9910
18	9.1683	1524	237.1330	0.1346	0.8654	4	656	0.0239	0.9908
19	8.3273	145.5289	28.1600	0.1621	0.8379	35	6871	0.0241	0.9906
20	1.2275	23.0060	3478	0.9934	0.0066	0.3	43467	00245	0.9908
21	4.9839	653.6816	214.1402	0.2468	0.7532	5	1530	0.0239	0.9908
22	94.2711	1857.1133	27.6413	0.0147	0.9853	36	538.5	00247	0.9901
23	1.2584	21.1318	590	0.9654	0.0346	2	47322	0.0245	0.9903
24	4.5057	63.7910	26.8675	0.2964	0.7036	37	15676	0.0239	0.9908

* The parameter values in the first row were obtained by Sprague et al. (2004). The experimental FRAP data are from J. McNally (personal communication).

**Figure 3:**
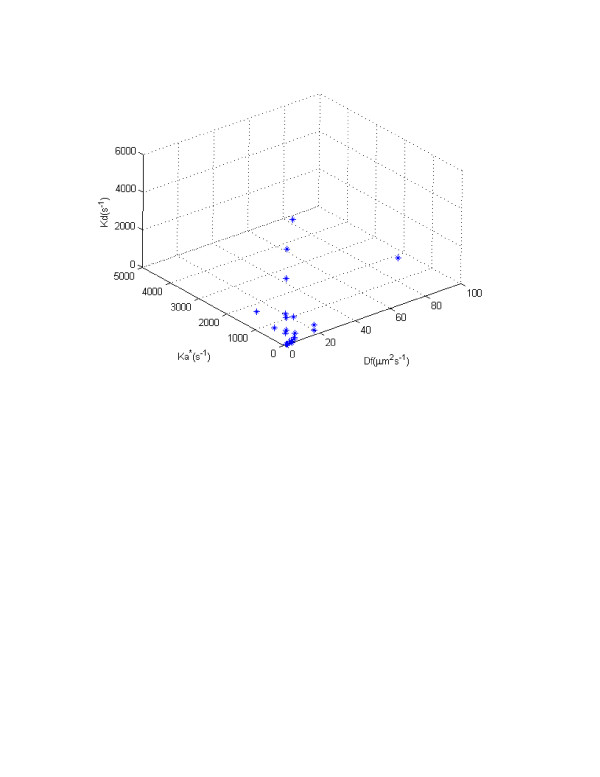
Three-dimensional parameter hyper-space.

To illustrate the non-uniqueness of the inversion, we plotted the optimized parameter values in three-dimensional parameter hyper-space (Fig. 3). The plot visibly indicates that the different combinations of model parameters can lead to same penalty (objective) function or error levels. The plot also suggests a potential linear form of correlation between bio-macromolecular diffusion coefficient and pseudo-association rate parameter. The first set of optimized parameters, obtained by [25], shows that 86% of the GFP-GR is bound to DNA, nuclear matrix or unknown binding sites and only 14% is free. Our analysis, however, suggests that using FRAP, one cannot conclude how much of the bio-macromolecule under study is free and how much is bound. As Table 1 shows, the concentration of free GFP-GR ranges from zero to 100 per cent. The same is true for the concentration of the bound complex. For example, referring to the results obtained in run 11, one may conclude that 100 per cent of the GFP-GR is free, while the results of run 6 show that all of it is bound. Note that both parameter sets produce excellent fits with the same *RMSE *and coefficient of determination (see Figure 2).

Analysis of Table 1 indicates that the average binding time per vacant site, calculated by *t*_*b *_= 1/*K*_*d *_[25], ranges between 0.3 and 500 mili-seconds. Similarly, the average time for diffusion of GFP-GR from one binding site to the next, obtained by *t*_*d *_= 1/$K_{a}^{*}$[46], varies over several orders of magnitude. The broad range of average binding time and average diffusion time for GFP-GR indicates that it is doubtful that one can infer an average time for macro-molecule diffusion and binding inside living cells from FRAP results alone.

This analysis explains the conflicting parameter values that may have been reported in literature for biomolecules using the FRAP protocol. Based on our findings, using experimental FRAP time series and coupling it with curve-fitting procedures can lead to misleading conclusions regarding binding reaction, slow or rapid mobility of biomolecules, and concentrations of free macromolecule, vacant binding sites and bound complex inside living cells and tissues.

In this study, the choice of a numerical approach rather than an analytic solution and a finite element approach rather than the finite difference scheme was made so that the parameter estimation could be readily extended to arbitrary initial and boundary conditions, complex domain geometry, and especially so that it could be extended to a ternary system of coupled nonlinear partial differential equations governing transport of free bio-macromolecule, bound complex, and vacant binding site where all three transport entities are moving species and it is impossible to obtain an exact solution for the systems of equations.

So far most of the FRAP studies have assumed an infinite domain to specify boundary conditions and to solve the system of partial differential equations governing biomolecule transport in-vivo [[13,25,29,30] among many others]. This assumption is unrealistic in the context of living cells. In this study, we address and improve this shortcoming by specifying a finite domain and by formulating Neumann boundary conditions on the cell membrane.

### Residual analysis

The inverse methodology used in this study is based upon the following assumptions: 1) residuals have a mean of zero, 2) residuals have constant variance, 3) residuals are uncorrelated, and 4) residuals are normally distributed. When these assumptions are met, the parameter optimization estimates poses optimal statistical properties [40-42]. When these conditions are not met the parameter optimization method *may *no longer produce optimal parameter estimates. Residuals, or errors in parameter optimization, are defined as the difference between the observed and simulated state variable(s). An analysis of the residuals is a useful and key technique to study possible trends, oscillations, and correlation of errors. It is also important in validating the assumptions on which the inverse modeling strategy rests.

To analyze the residuals, they were plotted against average normalized fluorescence intensity, $\bar{I}$(*t*), within the bleach spot during the time course of the FRAP experiment. Since the residuals are time and/or space series, their possible correlation was thoroughly analyzed. Different statistical measures such as error frequency analysis, normal probability plot, and hypotheses tests were explored to make decision about the residuals. The *Student's t-test *was used to test if the residuals have a mean of zero. *Bartlett's test *[47] was applied to determine if the residuals have constant variance. To test the normality of the residuals the Chi-square and Kolmogorov-Smirnov one sample tests were employed. Finally, the *t-statistic *[48] was used to test if the residuals are correlated.

The basic assumption in the *hypothesis test on the residuals' mean *is that the data come from a normally distributed population with unknown variance. In this study, the following *null and alternative hypotheses *were formulated:

(17) $$\begin{array}{l} H_{0}:\mu =\mu_{0}\\ H_{A}:\mu \neq \mu_{0} \end{array}$$

To perform the test the following *critical t-statistic *(*t*) was used:

(18) $$t=\frac{\bar{x}-\mu_{0}}{s/\sqrt{N}}$$

in which $\bar{x}$, *s*, and *N *are the mean, standard deviation, and size of the sample (errors), respectively. *μ*_0 _is the mean of the population which is zero.

For -*t*_*α*/2 _<*t *<*t*_*α*/2 _the null hypothesis (mean is zero) cannot be rejected at the significance level of *α*. The rejection regions *t *< -*t*_*α*/2 _or *t*_*α*/2 _<*t *indicate that the null hypothesis can be rejected at the level of significance *α*.

The mean and standard deviation of the residuals were -0.0029 and 0.0234 with sample size *n *= 43. The *t-statistic *was calculated as:

$$t=\frac{-0.0029-0}{0.0234/\sqrt{43}}=-0.8127$$

For 42 degrees of freedom, the *tabled t-values *for different levels of significance are given in Table 2. The calculated *t-statistic *was then compared with the *tabled t-values *at different levels of significance and the results summarized in Table 2. As the Table indicates the *null hypothesis *(mean of the residuals is zero) can not be rejected even at 20 per cent level of significance. The possibility of committing *error type one *is extremely slim.

**Table 2:** The results of hypothesis test on the residuals' mean in FRAP model.

*α*	0.01	0.05	0.1	0.2
*t-value*	2.8120	2.0175	1.6820	1.3020
Decision	*Accept H*_0_	*Accept H*_0_	*Accept H*_0_	*Accept H*_0_

The *Bartlett's test statistic *was used to verify for equality of variances across sub-groups of a sample against the alternative that variances are not constant. Equal variance across samples is called *homogeneity of variances *and is usually used in several statistical tests such as analysis of variance and nonlinear optimization which assumes that the errors have constant variance [47]:

(19) $$T=\frac{(N-k)\ln s_{p}^{2}-\sum_{i=1}^{k}(N_{i}-1)\ln s_{i}^{2}}{1+\frac{1}{3(k-1)}\left[\sum_{i=1}^{k}\frac{1}{N_{i}-1}-\frac{1}{N-k}\right]}$$

where $s_{i}^{2}$ is the variance of the subgroup, *N*_*i *_is the sample size of the subgroup, *k *is the number of subgroups, and $s_{p}^{2}$ is the pooled variance. This variance is a weighted average of the variances:

(20) $$s_{p}^{2}=\sum_{i=1}^{k}(N_{i}-1)\ln s_{i}^{2}/(N-k)$$

The rejection region is those values of *T *> $\chi_{\left(\alpha ,k-1\right)}^{2}$ in which $\chi_{\left(\alpha ,k-1\right)}^{2}$ is the *upper critical value *of the *chi-square distribution *with *k *- 1 degree of freedom at the level of significance *α*.

The following *null and alternative hypotheses *were formulated:

(21) $$\begin{array}{l} H_{0}:\sigma_{1}^{2}=\sigma_{2}^{2}\\ H_{A}:\sigma_{1}^{2}\neq \sigma_{2}^{2} \end{array}$$

To verify if the residuals have constant variance they were divided into different sections. One of the possible solutions in Table 1 was chosen and the residual plot versus laser beam recovery (Fig. 3) was divided into three regions. The variance in each region was calculated and compared with each other using the Bartlett test. The residuals were divided into three groups as:

$$S_{1}^{2}$$

= *S*^2^(*r*(1:5)) = 9.2651 × 10^-4^

$$S_{2}^{2}$$

= *S*^2^(*r*(6:24)) = 9.3655 × 10^-4^

$$S_{3}^{2}$$

= *S*^2^(*r*(25:33)) = 9.3126 × 10^-5^

The pooled weighted variance was found to be $S_{p}^{2}$ = 7.1030 × 10^-4^. The *Bartlett's statistic *was calculated as *T *= 9.5454 which is less than the *upper critical value *of the *χ*^2 ^for two degrees of freedom (*k *= 3) at one per cent level of significance ($\chi_{\left(0.01,2\right)}^{2}$ = 10.60). It is, however, more than the tabled value for five per cent level of significance. At one percent level of significance the *null hypothesis *(the residuals have constant variance) cannot be rejected. Based on this test and analysis of residual plot versus laser beam recovery, it is concluded that the residuals have equal variance.

The following *null and alternative hypotheses *were used to test possible correlation among the residuals:

(22) $$\begin{array}{l} H_{0}:\rho =0\\ H_{A}:\rho \neq 0 \end{array}$$

where *ρ *is the correlation coefficient in the population. For *n *> 2 these hypotheses can be tested using the following *t-statistic *[48]:

(23) $$t=\frac{r_{s}}{\sqrt{\frac{1-r_{s}^{2}}{N-2}}}$$

in which *r*_*s *_is the correlation coefficient in the sample.

The null hypothesis (correlation coefficient is zero) is rejected when the absolute value of the *t-statistic *is greater than the *critical t-value *(*t *< -*t*_*α*/2 _or *t*_*α*/2 _<*t*) at the level of significance *α*.

The residuals were first divided into two sub-groups:

*r*_1 _= *r*(1 : *end *- 1)

*r*_2 _= *r*(2 : *end*)

The correlation coefficient (*r*_*s *_= 0.1931) was then calculated and was used to obtain the *critical t-statistics *(with sample size *n *= 42):

$$t=\frac{0.1931}{\sqrt{\frac{1-0.0373}{42-2}}}=1.2447$$

These *critical t-statistics *were then compared with the *tabled t-values *at different levels of significances and the results presented in Table 3. As the Table indicates the *null hypothesis *(residuals are uncorrelated) can not be rejected even at 20 per cent level of significance. The possibility of having *error type one *is almost zero and, therefore, we didn't perform autocorrelation/serial correlation analysis.

**Table 3:** The results of hypothesis test on the correlation of residuals in FRAP model.

*α*	0.01	0.05	0.1	0.2
*t-value*	2.7040	2.0210	1.6820	1.3030
Decision	*Accept H*_0_	*Accept H*_0_	*Accept H*_0_	*Accept H*_0_

One of the assumptions of the least squares theory, which was applied in this study, is the normality of the residuals. In other words, it is assumed that the errors are normally distributed. To analyze the normality of the errors, two qualitative and two quantitative methods were used: 1) Error frequency analysis and normal probability plots, and 2) Hypothesis tests on the normality of the residuals using *the chi-square goodness of fit test*, which is based on the differences between the observed (*o*_*i*_) and expected (*e*_*i*_) error frequencies will be used [49]:

(24) $$\chi^{2}=\sum_{i=1}^{k}\frac{{\left(o_{i}-e_{i}\right)}^{2}}{e_{i}}$$

where *k *is the number of intervals or cells.

**Figure 4:**
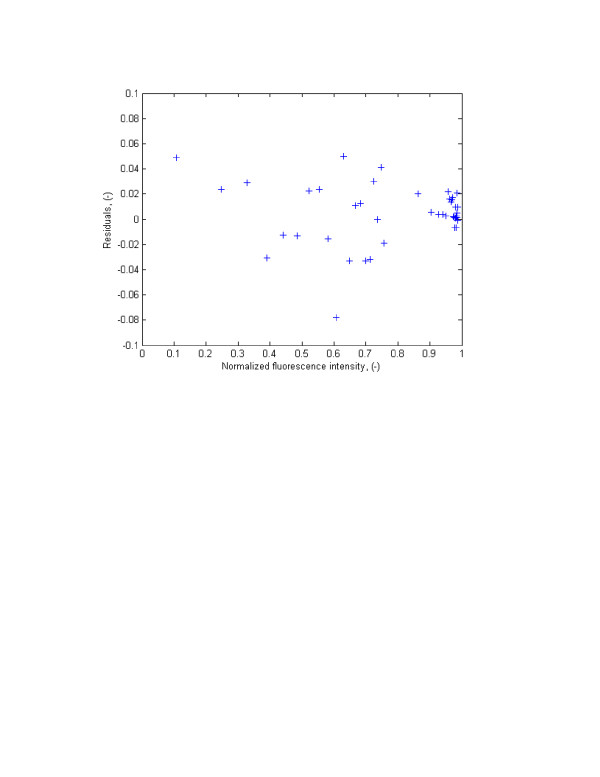
Residuals versus normalized average fluorescent intensity in FRAP experiment using one-site-mobile-immobile model.

**Figure 5:**
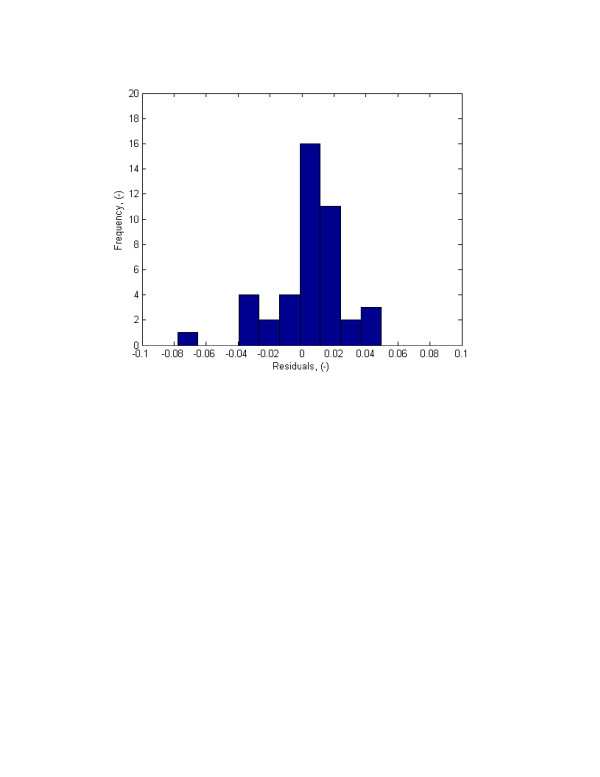
Histograms of residuals for normalized average fluorescent intensity using one-site-mobile-immobile model.

Error frequency analysis was first performed by constructing residuals' histogram. The histogram is presented in Figure 4. The Figure visibly shows that the errors are normally distributed. This was confirmed by the analysis of the normal probability plot (Fig. 5) and the *chi-square hypothesis *test on the normality of the random variable.

Residual frequency analysis and normal probability plots, though useful in figuring out the underlying probability distribution function, are only qualitative means to study possible normal distribution of random variable. To verify normality of the errors, the chi-square test was used. To perform the test, first the residuals were grouped into different cells (histograms). The number of residuals in every cell were counted which is *e*_*i*_. Then using the upper limit of the cells, the mean, standard deviation, and the cumulative normal distribution, the expected frequencies were calculated. The cells were merged when the observed error frequencies were less than 5. Then the *χ*^2 ^index was calculated and compared with $\chi_{\left(1-\alpha ,v\right)}^{2}$[50]. This information was used in the hypothesis test. The null and alternative hypotheses were formulated as:

(25) $$\begin{array}{l} H_{0}:r=N(\mu ,\sigma )\\ H_{A}:r\neq N(\mu ,\sigma ) \end{array}$$

or;

*H*_0 _: *r *= *N*(0.0012,0.0224)

*H*_*A *_: *r *≠ *N*(0.0012,0.0224)

where *μ *and *σ *are the mean and the standard deviation of the residuals in population.

Since the calculated *χ*^2 ^(3.6190) is less than the *tabled value *($\chi_{\left(0.80,2\right)}^{2}$ = 4.61), the *null hypothesis *(the residuals are normally distributed) cannot be rejected even at 20 per cent level of significance implying that the residuals are normally distributed.

The *chi-square test *is a powerful test when the sample size is large. However, combining cells when the expected error frequencies are less than five loses information and hence decreases the power of the test. Furthermore, for very small samples this test is not applicable [49]. To overcome these limitations, the Kolmogorov-Smirnov one sample test is usually used since it treats each observation separately and does not lose information through merging of categories. This test is more powerful than the chi-square test when sample size is not large.

The Kolmogorov-Smirnov one sample test was used to verify if the residuals are normally distributed. Results (not shown here) indicate that *the null hypothesis *(the errors are normally distributed) cannot be rejected even at 20 per cent level of significance implying that the residuals are strongly normally distributed.

In conclusion, detailed residual analysis indicates that: 1) residuals have zero mean, 2) residuals have constant variance, 3) residuals are normally distributed, and 4) residuals are uncorrelated. Therefore, the necessary and sufficient criteria for least square parameter optimization, which was used in this study, were met.

## Conclusion

A Galerkin-based finite element model was developed and applied to solve a system of two coupled partial differential equations governing GFP-GR transport and reaction in living cells. A finite domain was used to formulate boundary conditions on the cell membrane. The simulator was coupled with the Levenberg-Marquardt algorithm and a FRAP time series to estimate protein mass transport and reaction rate parameters. The developed strategy presents excellent agreement with the experimental data and the developed strategy is an efficient approach to extract as much physiochemical information from the FRAP protocol as possible. Uniqueness analysis of the inverse problem indicates that the FRAP protocol provides insufficient information for unique quantification of diffusion coefficient and binding rate parameters.

## Competing interests

The author(s) declare that they have no competing interests.

**Figure 6:**
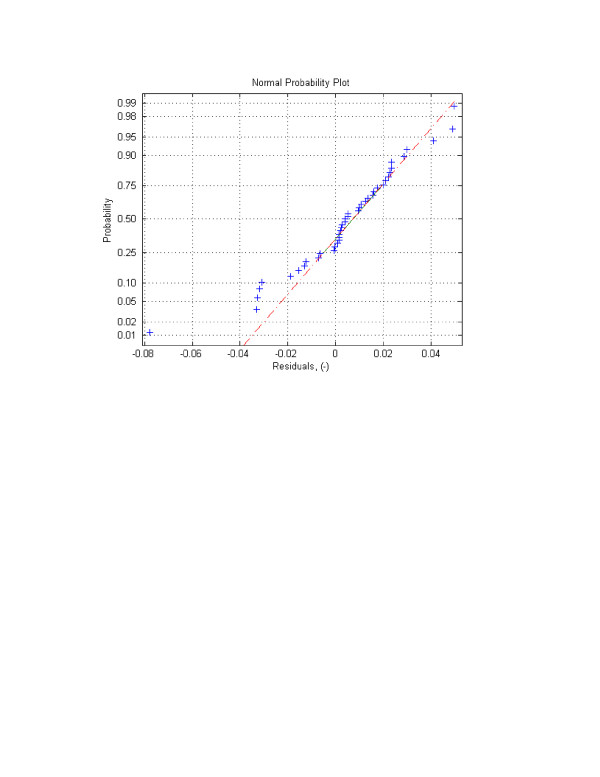
Normal probability plot for FRAP experiment using one-site-mobile-immobile model.
